# Does Social Feeding Improve Larval Survival of the Two-Spotted Lady Beetle, *Adalia bipunctata*?

**DOI:** 10.1673/031.012.10101

**Published:** 2012-08-25

**Authors:** Michael P. Moore, Charles R. Burt, Thomas D. Whitney, Steven A. Hastings, Gary C. Chang

**Affiliations:** ^1^ Biology Department, Gonzaga University, Spokane, WA 99258; ^2^ Watershed Studies Institute, Murray State University, Murray, KY 42071; ^3^ Great Basin Institute, Reno, NV, 89511; ^4^ Department of Entomology, University of Kentucky, Lexington, KY, 40546; ^5^ Department of Biological Sciences, Kent State University, Kent, OH 44242

**Keywords:** Aphididae, Coccinellidae, foraging behavior

## Abstract

Lady beetles typically lay eggs in clusters, and clutch-mates that emerge near to each other might benefit in multiple ways. For example, lady beetle larvae are attracted to the pheromone released by aphids under attack. Thus, one potential advantage to larvae emerging as a group is if one larva captures an aphid, others can share in consuming the same aphid. Sharing a meal likely reduces the per capita food intake of a hatchling, but it might also provide enough nutrition to prevent death by starvation during a particularly vulnerable stage. In an assay of the behavior of two-spotted lady beetles (*Adalia bipunctata*), larvae were attracted to chemical cues from damaged aphids, corroborating previous research. Densities of *A. bipunctata* hatchlings were then manipulated to test whether the presence of clutch-mates increasesed the probability of capturing prey, and the survivorship of hatchlings. In one experiment, a single aphid was placed with a number of lady beetle hatchlings ranging from 1 to 10 in a small arena for 72 hours to evaluate prey capture effectiveness and hatchling survival. As the initial density of lady beetle hatchlings increased, their prey capture rate increased. At the same time, survival of the hatchlings was not affected by their initial density. Five experiments were performed on individual fava bean plants by varying densities of aphids and lady beetle hatchlings to evaluate lady beetle survivorship measured after five days. In all five on-plant experiments, increasing the initial number of lady beetle larvae did not improve their survival. Lady beetle larvae shared meals during the small scale experiments, but that behavior did not improve their survivorship under any of the experimental conditions.

## Introduction

Newly hatched aphidophagous lady beetle larvae are inefficient at capturing prey, which makes their first meal particularly important. For example, Dixon (1959) observed individual unfed first instar *Adalia decempunctata* (Coleoptera: Coccinellidae) foraging for single aphids that were replaced if captured. Out of 50 larvae that were observed, 22 starved before capturing the first aphid. Of the 28 survivors, only 5 starved before capturing the second aphid. By laying eggs in clusters, female lady beetles are thought to improve the survival of their larvae in a variety of ways: availability of trophic eggs, sibling cannibalism, or by the “social feeding hypothesis” (Osawa 1989; Agarwala and Dixon 1993; Hemptinne et al. 2000). For example, fertile female lady beetles can increase the proportion of infertile “trophic” eggs they lay within a clutch when prey density is low (Perry and Roitberg 2005). Consumption of trophic eggs supplies some initial nutrition for newly hatched larvae. Similarly, the rate of sibling cannibalism increases as the density of larvae within a patch increases (Osawa 1989). Cannibalizing siblings increases survivorship and augments development within the first instar, although it may not sustain development into further stages (Snyder et al. 2000). One cost of sibling cannibalism is that it lowers the fitness of the mother when aphid density is high (Osawa 1992).

Hemptinne et al. (2000) developed the social feeding hypothesis as another, non-mutually exclusive, explanation of how laying eggs in clusters might improve larval survivorship. The premise for the social feeding hypothesis involves the chemical E-β-farnesene, that aphids will secrete when under attack from a predator and that functions as an alarm pheromone (Bowers et al. 1972). Lady beetles can use E-β-farnesene as an olfactory cue to locate aphids (Obata 1986; Hemptinne et al. 2000; Mondor and Roitberg 2000; Francis et al. 2004). Hemptinne et al. (2000) further showed that the movement of lady beetle larvae toward an aphid that has been captured by another larva can result in several larvae feeding on a single aphid. They then hypothesized that that larvae from clusters with many siblings are more likely to survive than larvae from smaller egg clusters due to the mutual olfactory attraction to captured prey, lessening the burden on the individual larva to make a successful capture. Hemptinne et al. (2000) wrote that a key prediction of the social feeding hypothesis needs testing, specifically that larvae hatching from eggs in clusters have a greater per capita chance of survival than larvae hatching from eggs laid singly.

The objectives of this study were to test for the presence of behavioral components that allow for social feeding to benefit larvae, and to test whether social feeding mitigates larval starvation in the two-spotted lady beetle, *Adalia bipunctata* L. (Coleoptera: Coccinellidae). Female *A. bipunctata* typically lay between 2 and 41 eggs in a clutch (Timms and Leather 2007), a range that makes them a good candidate for testing the social feeding hypothesis. This study had three primary components. First, the attraction of *A. bipunctata* larvae to olfactory cues from aphids and aphids being attacked by larvae was assessed. Second, the effect of differing numbers of larvae on aphid capture rate was tested in small experimental arenas. Finally, experiments that manipulated the density of *A. bipunctata* larvae were performed on individual fava bean plants stocked with aphids. Larval survival was the primary response variable in the on-plant experiments. If social feeding is an adaptation for alleviating starvation, then increasing initial larval density should increase larval survivorship.

## Materials and Methods

### Experimental Overview

All experiments were conducted between May 2010 and July 2011. Adult specimens *of A. bipunctata* were collected in Spokane, Washington from the Gonzaga University campus. This species is native to the Pacific Northwest region of the United States, and exhibits social feeding behavior (Hemptinne et al. 2000). Adult *A.* *bipunctata* were kept in the laboratory on a diet of pea aphids, *Acyrthosiphon pisum* (Harris) (Homoptera: Aphididae) *ad libitum*, and allowed to produce eggs in paper condiment cups (No. 075S, Solo Cup Co., Highland Park, IL). Organisms were kept at ambient room temperature (18–22° C), and relative humidity (32–45%) on a 16:8 L:D photoperiod during both routine rearing and the experiments described below. Three types of experimental arenas were used for the experiments that composed the study. In Experiment A, the response of *A. bipunctata* larvae to different olfactory cues was assessed in a Petri dish assay. In Experiment B, the capture rates of aphids by differing numbers of larvae were measured in small plastic cups. Finally, Experiments C-G on larval survivorship on individual fava bean plants, *Vicia faba* L. (Fabales: Fabaceae), were performed in tall glass vases. Statistical analyses were performed in R version 2.12.0 (R Development Core Team 2010).

### Experiment A: Olfactory Attraction Assay

**Figure 1.**
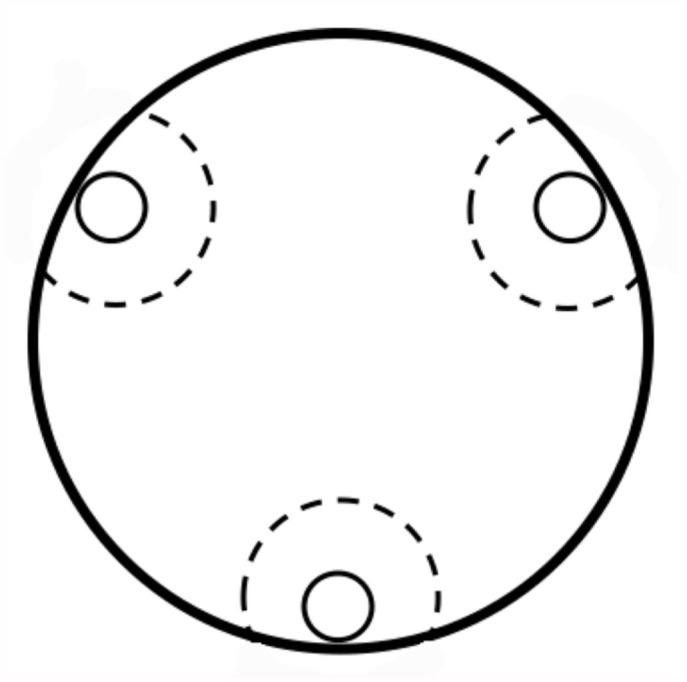
Experiment A. Schematic diagram of the arenas used for the olfactory attraction assay. The thick solid line represents the wall of the bottom of a 9 cm Petri dish. The thinner solid lines represent segments of a plastic straw with holes, into which one of three treatments was randomly placed. The straw segments were capped on each end with wax film. The dashed lines represent the boundaries between each treatment's “zone” and the background zone. High quality figures are available online.

The behavior of the population of *A. bipunctata* used in this study was evaluated within the bottoms of 9 cm Petri dishes that were left uncovered (Figure 1). The walls were coated with “Insect-a-Slip” (BioQuip Products, Inc., Rancho Dominguez, CA), and a 9-cm diameter piece of filter paper was pressed to the floor of each Petri dish. Using a pencil, three equidistant zones (3 cm in diameter) were traced at the edges of the filter paper. A plastic drinking straw (1.2 cm diameter) was cut into segments of 1.2 cm height. One of three treatments was placed inside each straw segment: (1) 2 crushed pea aphids, (2) 2 crushed pea aphids and 3 newly hatched *A. bipunctata* larvae, or (3) an empty segment control treatment. The ends of a straw segment were covered with Parafilm ® (American National Can, Menasha, WI). Eight holes were poked into the straws at 0.6 cm height to permit the dispersal of chemical cues from inside the straw. Assembly of an arena was completed when a straw segment was attached using Elmer's Glue-All (Elmer's Products, Inc., Columbus, OH) to the center of each of the three zones. Each treatment zone was present in an individual arena. In addition to the three zones that were centered on each straw segment, the remaining area of the arena was considered to be a fourth, background zone. At the start of a trial, a single newly hatched larva was placed in the center of the arena. The trial larva was allowed to walk freely for 30 minutes, and the amount of time it spent in each zone was recorded. Ten replicates were performed. The amount of time that the larvae spent in each zone was compared using compositional analysis of habitat use (Aebischer et al. 1993). Compositional analysis uses both the time spent in different areas and the sizes of the different areas to rank the preferences of animals for different locations. For this experiment, time spent in the background zone was used as the denominator for log-ratio transformations.

**Table 1.**
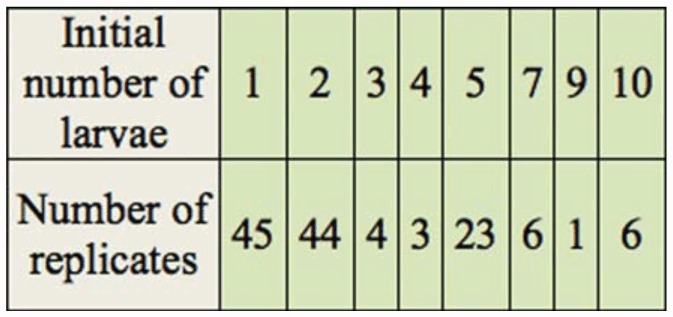
Experiment B, the small scale capture trials. Initial densities *of Adalia bipunctata* larvae and number of replicates.

### Experiment B: Small Scale Capture Trials

The effects of larval density on their ability to capture prey and on their early survival was tested in 30-mL plastic containers with lids (Solo Cup Co., Highland Park, IL). For these trials, newly emerged larvae from the same cluster of eggs were gently placed in a container using a moist fine paintbrush. Between 1 and 10 larvae were initially placed in a container (Table 1). Larvae were removed from their egg cluster to eliminate the opportunity for trophic egg consumption and cannibalism (Osawa 1989; Perry and Roitberg 2005). One third instar pea aphid was then placed in each condiment cup. After 24 hours, the aphid was recorded as either successfully captured by the lady beetle larva(e), or not captured. Survivorship of the *A. bipunctata* larvae was observed at 48 and 72 hours. The binary attribute variable of aphid capture success was analyzed using logistic regression with each container as a replicate, and the initial number of *A. bipunctata* larvae as the independent variable. The proportion of *A. bipunctata* larvae that survived in a replicate was a discontinuous measurement variable, with numerical values that were fixed based on the initial number of larvae in a replicate. The proportion of surviving larvae in a replicate was logit transformed to improve homoscedasticity (Warton and Hui 2011) and analyzed using linear regression with the initial number of larvae as the independent variable.

**Table 2.**
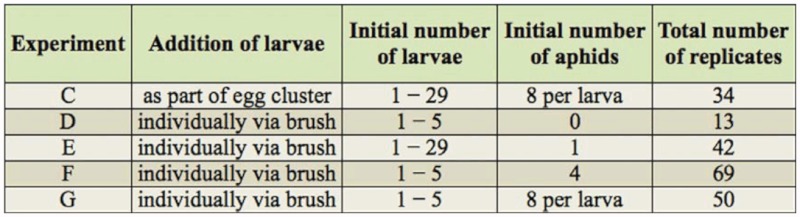
Summary of experiments conducted on individual fava bean plants. Experiments are referenced by letter in the text.

### Experiments C-G: Survival on Individual Plants

Five experiments testing the effect of initial larval density on per capita larval survival were conducted using individual potted fava bean plants as the experimental arena (Table 2). The five experiments differed in the density of pea aphids, density of larval *A. bipunctata*, and method of larval introduction. For these trials, fava beans were grown under greenhouse conditions until they had at least three expanded leaves. When a plant was large enough to be used for a trial, it was transferred into a tall glass vase, and insects were added. Finally, the vase was then topped with fine mesh. All *A. bipunctata* larvae used within an individual trial originated from the same egg cluster. Each trial lasted five days, after which the number of larvae that survived was counted.

Experiment C introduced freshly hatched *A. bipunctata* to a plant hosting an initial surplus of aphids, and was replicated 34 times. To provide larvae for this experiment, adult female *A. bipunctata* were kept inside paper condiment cups that provided a surface upon which they oviposited. Once larvae hatched from the eggs in a cluster, but prior to their dispersal, the area of cup holding the cluster of emerging larvae and unhatched eggs was cut from the remainder of the cup. The piece of cup holding the cluster was then glued to the underside of a leaf of the fava bean plant using Elmer's Glue-All. Then, the number of hatchlings and unhatched eggs was recorded. Pea aphids were added to the plant in a ratio of eight aphids per *A. bipunctata* hatchling. This treatment was designed to mimic the typical field conditions of adult female lady beetle oviposition, and subsequent larval dispersal (Wratten 1973). Multiple regression was used to evaluate whether the initial number of larvae and the proportion of eggs that hatched affected larval survivorship in the trials. The proportion of eggs that hatched and larval survivorship were logit transformed to better meet the assumptions of linear regression.

The remaining experiments examined *A. bipunctata* hatchlings that were separated from their egg cluster as they hatched, using a moistened fine paintbrush. Separation of larvae from eggs was done to ensure that they were unfed, and to eliminate the possible effects of trophic eggs and egg cannibalism. The numbers of total replicates in these experiments varied (Table 2), partially due to lower numbers of larvae being more commonly available to use in a trial than higher numbers of larvae. Other details of the experimental designs of Experiments D-G were altered slightly in order to focus on different factors that might affect larval survival. For Experiment D, between one and five larvae were added to plants without any aphids to examine whether newly emerged larvae could survive by tolerating starvation or avoiding larval cannibalism. For Experiment E, between 1 and 29 larvae were placed on plants that hosted a single aphid. For Experiment F, larvae were introduced to plants that hosted four aphids. In Experiment G, plants hosted eight aphids per larva to repeat the conditions of Experiment C, but in the absence of unhatched conspecific eggs. Data from Experiment E suggested that any benefit from social feeding would most likely occur when between one and five newly hatched larvae were on a plant. Therefore, for Experiments F and G, the initial number of larvae was restricted to between one and five. The proportion of larvae that survived to five days in a trial was logit transformed and analyzed using linear regression with the initial number of larvae as the independent variable.

## Results

### Experiment A: Olfactory Attraction Assay

**Figure 2.**
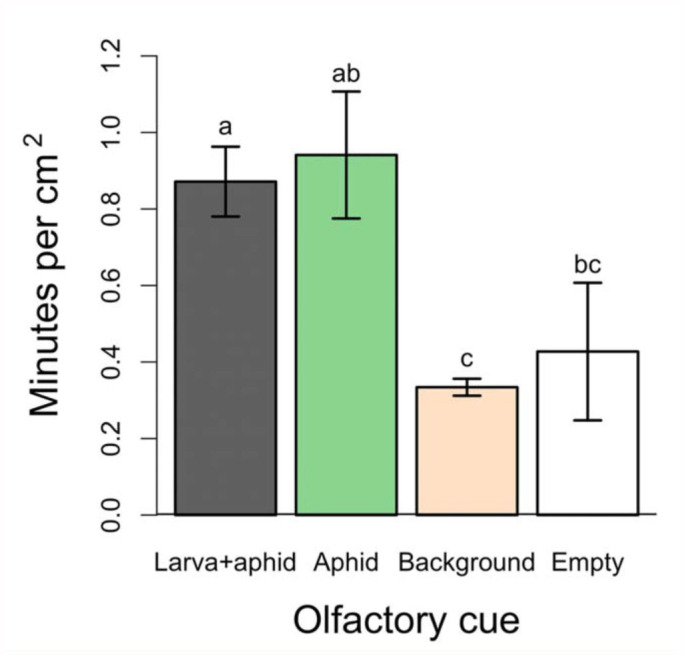
Experiment A. The attractiveness of various olfactory cues to first instar *Adalia bipunctata.* The amount of time spent in each area of the arena has been standardized by the size of the areas. Columns with different letters indicate responses that were significantly different (*p* > 0.05) in pairwise comparisons by compositional analysis (Aebischer et al. 1993). High quality figures are available online.

Larvae spent significantly different proportions of time in the different zones of the assay arena (*χ*2 = 15.519, df = 3, *p* = 0.001). The relative ranking of zones from most to least preferred was: aphids and larvae, aphids only, background, and empty. Pairwise comparisons of the time spent in the different zones found that larvae spent significantly more time in zones that contained aphids than in the background zone (Figure 2). The proportion of time spent by focal larvae in the zone containing aphids and larvae versus the zone containing only aphids did not significantly differ. Similarly, the proportion of time spent in the background versus the empty treatment zones did not differ.

### Experiment B: Small Scale Capture Trials

**Figure 3.**
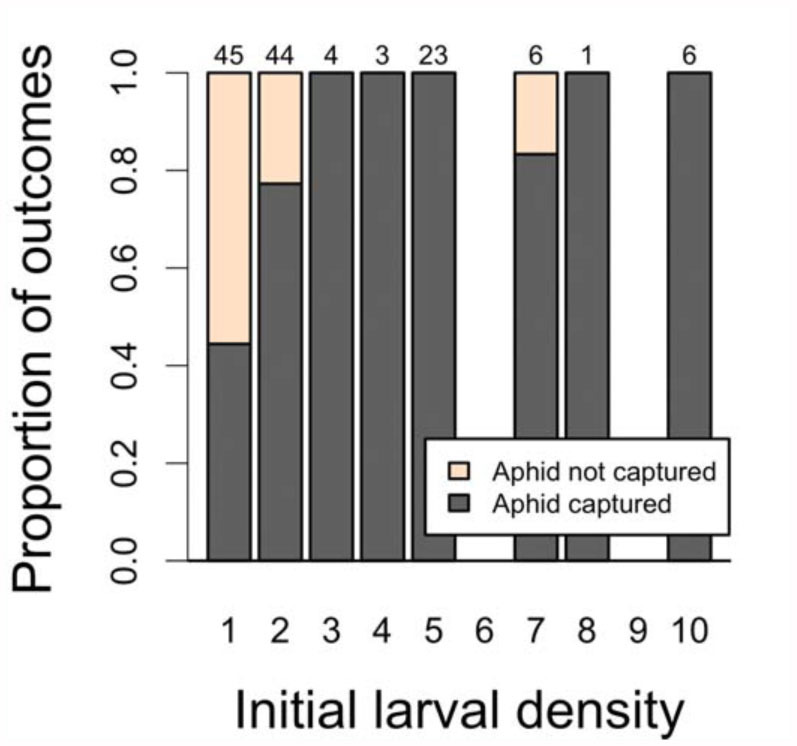
Experiment B. Proportion of trials resulting in successful and unsuccessful aphid capture versus the initial number of first instar *Adalia bipunctata.* Larvae were kept with a single aphid for 24 hours in a 30 mL container. The number above each column is the number of replicates at that initial larval density. High quality figures are available online.

**Figure 4.**
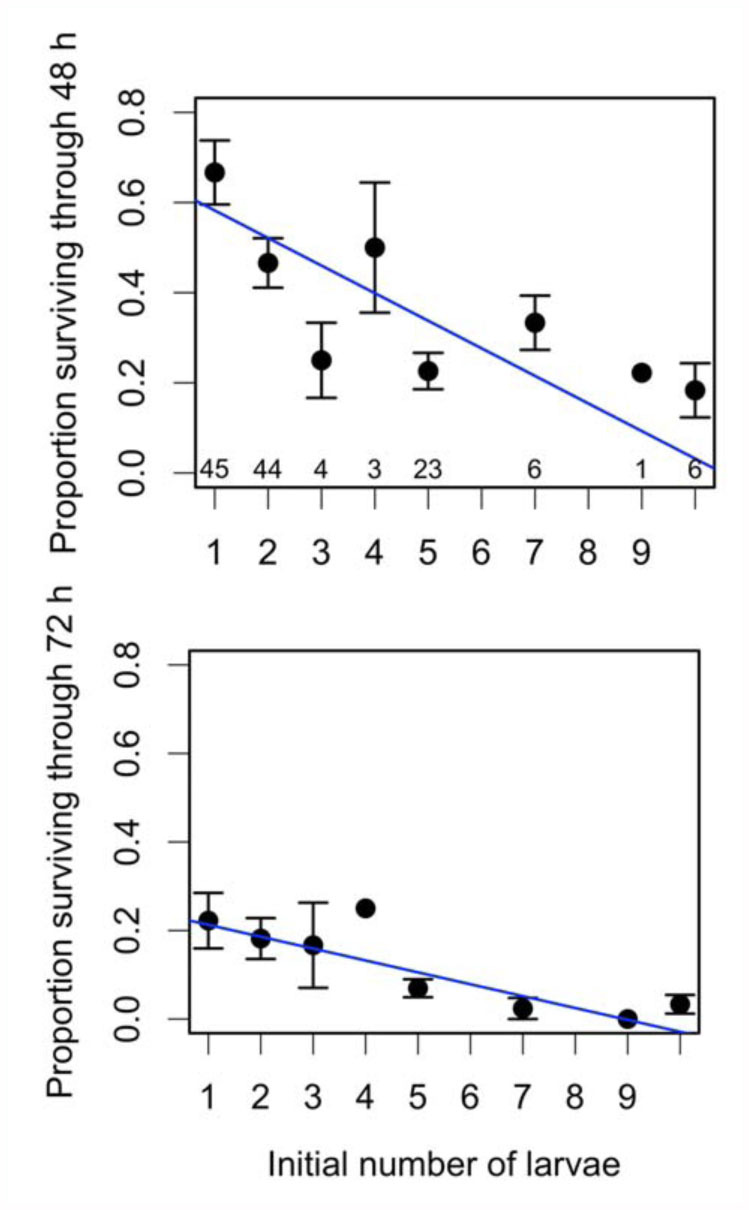
Experiment B. Survivorship of first instar *Adalia bipunctata* through (a) 48 hours and (b) 72 hours. Each symbol represents the mean of all replicates at an initial number of larvae, ± standard error. The number of replicates at each initial larval number is shown above the x-axis in panel a; the number of replicates is the same for panel b. The blue line represents the best fit line for each set of observations. High quality figures are available online.

A total of 383 larvae were tested in 132 trials in 30 mL containers. The proportion of trials in which the aphid was successfully captured within 24 hours increased when initial larval density increased in a cup (Figure 3; z_1,130_ = 3.506, *p* < 0.001). In 32 of 45 (71.1%) trials that tested a single larva, the aphid was captured within 24 hours. In contrast, when three or more larvae were tested, the aphid was captured in 42 out of 43 (97.7%) trials. Of the initial 383 larvae, 133 (34.7%) survived through 48 hours, and 42 (11.0%) survived through 72 hours. Larval survivorship through 48 hours decreased with initial larval density (Figure 4a; *F*1,130 = 15.78, *p* < 0.001). Initial larval density also decreased larval survivorship through 72 hours (Figure 4b; *F*_1.130_ = 4.191, *P* = 0.043).

**Table 3.**
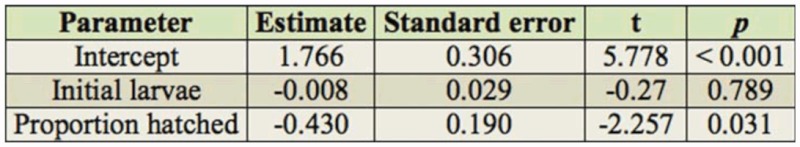
Experiment C. Multiple regression analysis of the influence of the initial number of larvae and the proportion of eggs that hatched from their cluster on the survivorship of *Adalia bipunctata* on fava bean plants. Plants were stocked with eight aphids per initial larva and larval survival was evaluated on the fifth day of each trial. Overall model statistics: multiple R^2^ = 0.198, F_(2, 31)_ = 3.815, *p* = 0.033.

### Experiments C-G: Survival on Individual Plants

**Figure 5.**
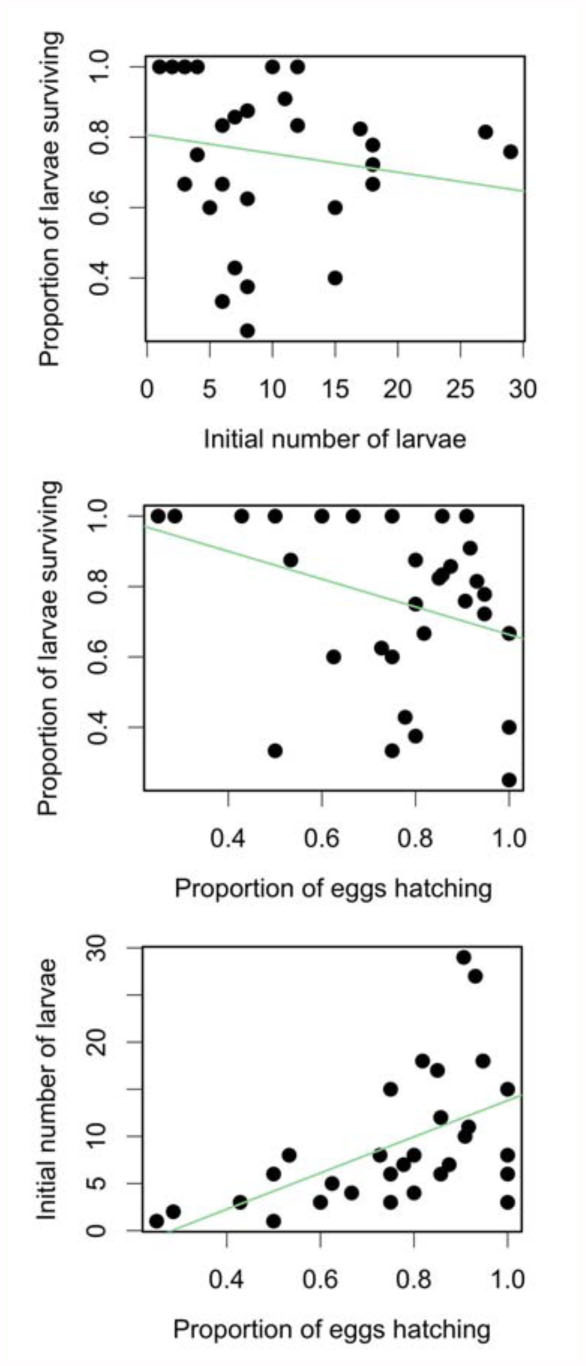
Experiment C. The relationship between survivorship of larvae to five days and initial number *of Adalia bipunctata* larvae (a), survivorship and proportion of eggs that hatched from an egg cluster (b), and initial number of larvae and proportion of eggs that hatched (c). Green lines represent the best fit line for each set of observations. Trials were conducted on a fava bean plant stocked with eight aphids per initial larva. Untransformed values are shown for survivorship and proportion of eggs that hatched; however, these variables were logit transformed for multiple regression analysis (Table 3). High quality figures are available online.

In Experiment C, larvae were allowed to remain on their egg cluster at the start of each trial. The number of eggs per cluster ranged from 2 to 32, and the mean number of eggs in the 34 replicates was 11.4. Out of 387 eggs used in the experiment, 312 (80.6%) hatched. Across all replicates, a total of 228 (73.1%) of the larvae survived to the fifth day. The proportion of eggs that hatched from a cluster was negatively associated with the survivorship of larvae to the fifth day (Figure 5, Table 3). The initial number of hatchlings in a trial had no relationship to their survivorship (Figure 5, Table 3).

**Figure 6.**
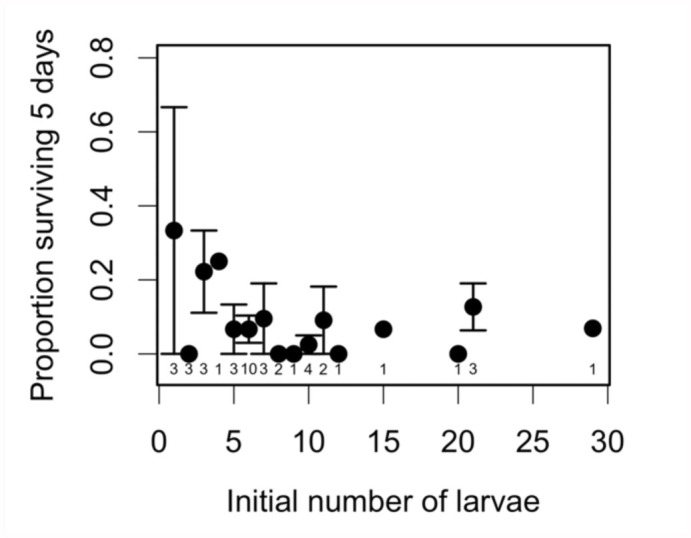
Experiment E. Survivorship of individually-transported larvae versus the initial number of larvae on plants stocked with a single aphid. The number of replicates at each initial larval number is shown above the x-axis. High quality figures are available online.

**Figure 7.**
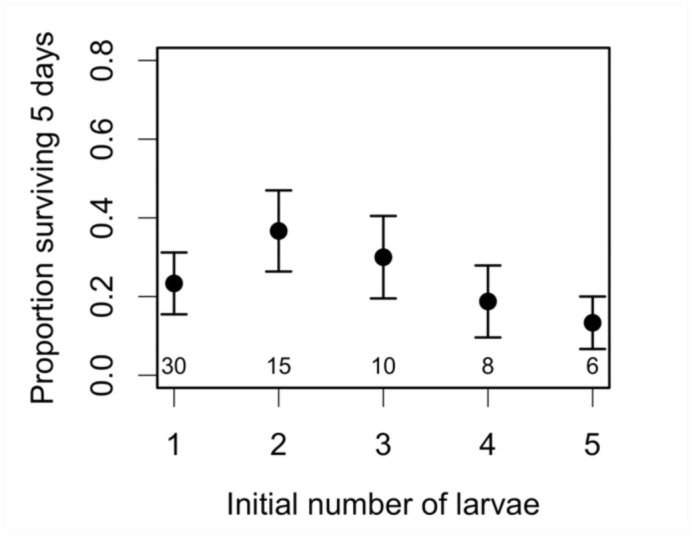
Experiment F. Survivorship of individually-transported larvae versus their initial number on plants stocked with four aphids. The height of each bar represents the mean of all replicates at an initial number of larvae, ± standard error. The number of replicates at each initial larval number is shown above the x-axis. High quality figures are available online.

**Figure 8.**
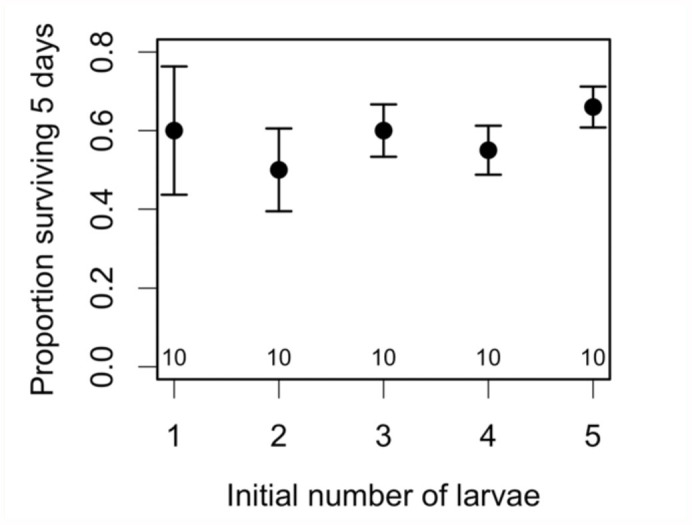
Experiment G. Survivorship of individually-transported larvae versus their initial number on plants stocked with eight aphids per larva. The height of each bar represents the mean of all replicates at an initial ± umber of larvae, ± standard error. The number of replicates at each initial larval number is shown above the x-axis. High quality figures are available online.

Newly hatched larvae were removed from their egg cluster in experiments D-G, so the proportion of eggs hatching from a cluster was no longer considered. In 13 replicates of Experiment D, without aphids, larvae never survived to the fifth day. In 42 replicates of Experiment E, where a single aphid was provided to the larvae, 25 out of 344 larvae survived until the fifth day (7.3%), and the number of larvae surviving within a replicate ranged from 0 to 4. When a single aphid was provided to the larvae, the initial density of larvae did not affect larval survivorship (Figure 6; *F*_1, 40_ = 0.228, *p* = 0.636). In 69 replicates of Experiment F where four aphids were provided to larvae, 37 out of 152 larvae survived until the fifth day (24.3%). The number of larvae surviving within a replicate ranged from 0 to 3. The initial number of larvae did not affect the proportion of initial larvae surviving to the fifth day (Figure 7; *F*_1, 67_ = 0.163, *p* = 0.688). Finally, in 50 replicates of Experiment G, where eight aphids were provided per larva, 89 out of 150 larvae survived until the fifth day (59.3%). Within a replicate, the number of larvae surviving ranged from 0 to 5. The initial number of larvae did not affect the survivorship of initial larvae on the fifth day (Figure 8; *F*1, _48_ = 0.172, *p* = 0.680).

## Discussion

The social feeding hypothesis of Hemptinne and colleagues (2000) connects behaviors observed in hatchling lady beetle larvae to a longer-term improvement in survival. In particular, the ability of first instar lady beetles to orient toward an aphid that has already been captured by a conspecific can allow multiple larvae to feed on the aphid, which leads to the prediction that lady beetle hatchlings that are part of a group should have higher survival rates than isolated larvae. Our findings provide another demonstration of lady beetle larvae being attracted to cues from damaged aphids. Furthermore, the results suggest that larger groups of larvae are more likely to capture an individual aphid. However, we did not find support for the prediction that being part of a group would improve the survival of larval lady beetles.

Our findings support previous work on the mechanistic basis for the social feeding hypothesis, which depends upon observations of coccinellid larvae using chemical cues to locate potential prey (Obata 1986; Hemptinne et al. 2000; Mondor and Roitberg 2000; Francis et al. 2004). In pairwise comparisons to controls (air that was free of aphid cues), *A. bipunctata* larvae oriented toward crushed aphids, and toward aphids being eaten by conspecific larvae (Hemptinne et al. 2000). In the current study, when larvae were given a three-way choice betweeen crushed aphids, crushed aphids with larvae, and a control, larvae preferred crushed aphids and crushed aphids with larvae equally (Experiment A; Figure 2). Thus, the presence of conspecific larvae did not alter the attractiveness of crushed aphids to first instar *A. bipunctata.* Multiple cues might have influenced larval behavior in this assay. For example, the larvae might have been attracted toward areas with greater relative humidity, as has been documented in other insects (Rebora et al. 2007). Another limitation of the assay method used was that test larvae did not come into direct contact with surfaces that had been previously walked upon by other larvae. While first instar *A. decempunctata* do not appear to avoid their tracks (Dixon 1959), trails left by larval lady beetles deter conspecific larvae on certain substrates (Rutledge et al. 2008).

Having more larvae in an area should increase the chance of any one larva encountering and capturing an aphid. In Experiment B, the probability that an aphid would be captured within 24 hours quickly approached 100% as the initial number of *A. bipunctata* in a trial increased (Figure 3). During observations of the trials before the 24 hour checkpoint, larvae that were not involved in the capture of the aphid were occasionally observed to orient toward a captured aphid and share in consuming it. However, the survivorship of larvae through 48 hours decreased as the number of larvae in a trial increased (Figure 4). We suggest that lower proportions of larvae survived in trials with more larvae because individual larvae were unable to consume enough of the aphid to avoid starvation, thus negating the benefit of social feeding.

The highly simplified arenas used in Experiment B might have obscured important factors allowing social feeding to improve the survival of *A. bipunctata* larvae. First, because only a single aphid was supplied in each arena and the number of capture successes was high, it was difficult to isolate a larval density that could most promote social feeding. Similarly, because the probability of capturing one aphid cannot exceed 100%, testing for a potential curvilinear (non-additive) effect was impaired. Finally, plant surfaces can provide additional cues that affect the outcomes of interactions between lady beetle larvae (Rutledge et al. 2008). Thus, Experiments C-G on individual plants were used to further examine the effects of the initial density of larvae on their survivorship.

Tests using a variety of aphid densities on fava bean plants failed to support the hypothesis that social feeding behavior would improve the survivorship of hatchling *A. bipunctata.* Larval survivorship was highest in Experiment C, where there were eight aphids per larva, and unhatched eggs accompanied the hatchlings. In Experiment C, the number of hatchlings leaving a cluster did not influence their survivorship (Table 3). In Experiment E, with a single aphid on a plant, an average of 7% of initial larvae survived. Again, the initial number of larvae had no significant effect on per capita larval survival (Figure 6). It is possible that a single aphid on a plant was too difficult to locate for low numbers of larvae, and provided too little nutrition for larvae at high densities (Nudds 1978; Mittelbach 1984). Additional aphids were provided to test larvae in Experiments F and G, and the initial number of larvae was limited to between one and five per plant. These conditions provided smaller numbers of larvae a greater chance of finding prey. Even within a set of conditions that was restricted to being more conducive to social feeding and offering a greater per capita amount of prey, no relationship was observed between initial larval density and survivorship (Figure 7, 8).

While this study emphasized testing the social feeding hypothesis, cannibalism and trophic egg consuption could have influenced the results. Numerous animals are more likely to turn to cannibalism when access to prey decreases (Polis 1981; Osawa 1989; Wagner and Wise 1997; Lucas et al. 1998). Previous work has found that when coccinellids are provided conspecific larvae to consume, they still require nutrition from aphids for long-term growth and survival (Snyder et al. 2000). In the current study, it was not possible to distinguish whether larval mortality was due to starvation or cannibalism, as the bodies of the larvae were difficult to distinguish from the potting medium. However, larvae lacked aphids, and had opportunities for posteclosure cannibalism in Experiment D, but no larvae survived to the fifth day of a trial. Therefore, aphid consumption was likely necessary for larvae to have survived for five days in Experiments E, F, and G. The developmental rate of larvae was not recorded in our study. Beyond the duration of our study, the potential effect of social feeding on variation in individual development might interact with post-eclosure cannibalism. By reducing variation in the food intake of individual larvae, social feeding could increase the level of synchrony in the developmental times of individuals in a cohort. Synchronized development would produce greater similarity in size among individual larvae. When juveniles of other cannibalistic arthropods are increasingly similar in size, they are less likely to engage in cannibalism (Hopper et al. 1996, Samu et al. 1999, Vanacker et al. 2004).

Lady beetle larvae frequently consume both trophic eggs and unhatched siblings, which can prolong their survival (Banks 1955; Perry and Roitberg 2005; Roy et al. 2007). Experiment C supplied hatchlings with unhatched eggs in addition to aphids as prey. The unhatched eggs were likely to have been consumed by at least some hatchlings, thus reducing their risk of starvation. The negative association between the proportion of eggs that hatched and the survivorship of hatchlings in this experiment (Table 3) is consistent with previous work that found that female lady beetles can alleviate much of the risk of larval starvation by increasing the number of trophic eggs in a cluster (Perry and Roitberg 2005).
